# Electrocardiographic Characteristics of Breast Cancer Patients Treated with Chemotherapy

**DOI:** 10.1155/2020/6678503

**Published:** 2020-12-09

**Authors:** Xufei Liang, Yueying Wang, Xi Yin, Xiaohong Gong, Shuo Pan, Ziliang Chen, Xuhong Geng

**Affiliations:** ^1^Department of Function, Fourth Hospital of Hebei Medical University, Shijiazhuang 050011, China; ^2^Graduate School of Medicine, Tianjin Medical University, Tianjin 300070, China

## Abstract

**Introduction:**

Patients receiving chemotherapy for breast cancer may be at risk of developing cardiac dysfunction and electrophysiological abnormalities. The aim of this study is to evaluate alterations in electrocardiographic (ECG) parameters in breast cancer patients receiving chemotherapy.

**Materials and Methods:**

This was a prospective single-center cohort study conducted in the Fourth Hospital of Hebei Medical University, China. Participants with breast cancer referred for chemotherapy from May 1, 2019, to October 1, 2019, were invited to participate in the study. Standard 12-lead ECG and echocardiography were performed at baseline or before chemotherapy (prechemotherapy) (T0), after 1 cycle (T1), after 3 cycles (T2), and at the end of chemotherapy (T3).

**Results:**

A total of 64 patients with diagnosed breast cancer undergoing chemotherapy were included. Echocardiographic parameters showed no significant variation during the entire procedure (all *P* > 0.05). The incidence of abnormal ECG increased from 43.75% at baseline to 65.63% at the end of chemotherapy, of which only the prevalence of fragmented QRS (fQRS) was significantly increased after the drug regimen (26.56% to 53.13%). At the end of the treatment, heart rate, P-wave dispersion, corrected QT interval, T-peak to T-end, RR, SV1, RV5, Sokolow–Lyon index (SLI), and index of cardioelectrophysiological balance deteriorated markedly (all *P* < 0.05). The area under the curve for SLI and QT dispersion (QTd) derived by ECG was 0.710 and 0.606, respectively. The cutoff value with 2.12 of SLI by ECG had a sensitivity of 67.2% and specificity of 71.9% for differentiating patients after therapy from baselines. The cutoff value with 0.55 of QTd had a sensitivity of 60.9% and specificity of 60.9%.

**Conclusions:**

The current study demonstrated that ECGs can be used to detect electrophysiological abnormalities in breast cancer patients receiving chemotherapy. ECG changes can reflect subclinical cardiac dysfunction before the echocardiographic abnormalities.

## 1. Introduction

One of the important side effects of chemotherapeutic agents used in patients with breast cancer is cardiotoxicity, which refers to cardiac dysfunction and heart failure [1]. Anti-HER2 agents and chemotherapies (specifically anthracyclines, which are frequently used to treat HER2+ breast cancer) have been associated with increased risk of cardiotoxicity [2, 3]. As treatment efficacy increases, there is an increasing number of patients who survive for extended periods and may receive chemotherapies for longer durations. Therefore, cancer patients increasingly require long-term management of chemotherapy-related morbidities. It is imperative to detect chemotherapy-induced cardiac injury in the early stage in order to, with the help of early pharmacologic intervention, prevent the occurrence of clinical heart failure. It has been reported that standard 12-lead electrocardiogram (ECG) enables the detection of different findings of cardiotoxicity such as sinus tachycardia, ST-T wave abnormalities, cardiac conduction disorders, QT prolongation, fragmented QRS, and cardiac arrhythmia during chemotherapies in cancer patients [1, 4, 5]. The 12-lead ECG remains a routine screening tool owing to its noninvasive, rapid, and inexpensive properties, and it has demonstrated promise as a tool for measuring subclinical cardiotoxicity [6]. The identification of patients at risk for cancer therapy-induced malignant arrhythmias is of exceptional clinical importance.

Previous studies have mainly focused on global left ventricular function changes during chemotherapy. However, in fact, the administration of chemotherapeutic agents may affect the cardiac electrophysiological properties before significant mechanical impairment. Therefore, we aimed to evaluate the presence or absence of ECG abnormalities in patients newly diagnosed with breast cancer following chemotherapies.

## 2. Materials and Methods

### 2.1. Study Population

In total, 64 eligible female patients with early-stage breast cancer were included in this single-center, prospective observational clinical study between May 2019 and December 2019. 35 patients had left-sided breast cancer, and 29 patients had right-sided breast cancer. All patients received adjuvant chemotherapy after breast cancer surgery. The exclusion criteria were age under 18 years or over 80 years, other malignancies, a previous history of chemotherapy and radiation therapy (RT), pregnancy or breastfeeding, acute myocardial infarction within the previous 6 months, symptomatic heart failure (New York Heart Association Functional Classification III-IV), left ventricular ejection fraction (LVEF) <50%, structural heart disease, serious cardiac arrhythmias, chronic use of drugs known to induce cardiac damage or arrhythmia, dialysis, permanent anticoagulation, and severe psychiatric disorders life expectancy less than 6 months. The study complied with the Helsinki Declaration, and the local institutional board of ethics approved the protocol. All participants signed informed consent before enrolment. Fourth Hospital of Hebei Medical University Research Ethics Committee approved the protocol (2020011).

### 2.2. Echocardiography

Echocardiography was performed by a cardiologist with experience in advanced echocardiography and trained for the requirements of the study, using standard parasternal and apical views with the frame rates of 45–75 frames/s and a GE Vivid E9 ultrasound system (GE Vingmed Ultrasound, Horten, Norway) equipped with a 2.0–4.5 MHz transducer and following current recommendations for cardiac chamber quantification in adults. Echocardiography data were collected from the department of function database [7–9]. Echocardiography was performed by the same cardiologist, who was blinded to the clinical data and electrocardiographic data.

### 2.3. Electrocardiography

Twelve-lead ECGs were recorded before the chemotherapy for breast cancer was started at the resting and supine position (filter: 45 Hz, alternating current filter: 50 Hz, paper speed: 25 mm/s, and amplitude 10 mm/mV; Huanan Medical, Zhengzhou, China). All of the ECGs were transferred to a personal computer to decrease error measurements and then used for 400% magnification by Adobe Photoshop software. All of the measurements were performed on the screen by manual method. No patient had fewer than nine measurable leads, and all precordial derivations were included in the measurements.

The following automated ECG measurements were extracted: heart rate (HR), P-wave amplitude (PWA), QT interval (QTI), RR interval (RR), corrected QT interval (QTc), QRS duration (QRSD), PR interval (PRI), QRS axis, and index of cardioelectrophysiological balance (iCEB: QT/QRS [10]). The following variables were manually measured: P-wave dispersion (Pd), QT dispersion (QTd), and T-peak to T-end (TpTe). ST-T changes were analyzed according to the criteria of parameter measurement, and ECG diagnosis is based on the recommendation of the American Heart Association (AHA) (AHA/ACCF/HRS, 2007–2009) [11]. Criteria for ST-T changes were any of the following: (1) ST-segment abnormalities: the ST segment was measured at 80 ms after J point, and the meaningful change was described as ST-segment depression ≥0.05 mV, or ST-segment elevation ≥0.10 mV in the limb leads and/or ≥0.20 mV in the chest leads. (2) T-wave changes: (a) high and sharp T-wave: the peak of T-wave was >0.5 mV in the limb leads and/or >1.5 mV in the chest leads; (b) low and flat T-wave: the peak of T-wave was <0.1 mV in the limb leads or <0.2 mV in the chest leads; (c) bidirectional T-wave; and (d) inversed T-wave (inversion depth ≥0.1 mV). Fragmented QRS (fQRS) is defined as the presence of an additional R-wave (R′), R-wave, or the S-wave notching, or the presence of more than one R′-wave in two consecutive leads [12]. An ECG is classified as abnormal if the following features were detected: sinus arrhythmia, atrial fibrillation, premature atrial or ventricular contraction, atrioventricular block, fQRS, ST segment, or T-wave changes. ECG parameters of the patients were measured by two blinded independent cardiologists (Y. W and Z. C), and ECGs were evaluated by a third independent reviewer (X. G) when there was a discrepancy between the evaluations of the two readers. For each study patient, these values were calculated on average three times.

### 2.4. Statistical Analysis

Continuous variables were summarized by the median and interquartile range or mean ± standard deviation and compared by one-way analysis of variance (ANOVA) or Fisher's exact test; otherwise, median and interquartile range (IQR) were reported. Categorical variables were expressed as frequencies and percentages and compared using the chi-square tests. The area under the receiver operating characteristic (ROC) curve was calculated to determine the capability of various ECG parameters to discriminate patients after chemotherapy from baselines. IBM SPSS Statistics 22.0 (IBM Corp. Released 2013. IBM SPSS Statistics for Windows, version 22.0. Armonk, NY: IBM Corp.) was used for statistical analyses. A *P* value of <0.05 was considered significant.

## 3. Results

### 3.1. Baseline Clinical Characteristics of Study Population

The study enrolled 64 women (mean age, 49.09 ± 9.61 years) with breast cancer treated with chemotherapy. The mean body mass index was 24.02 ± 3.18 kg/m^2^. Among comorbidities, diabetes mellitus was present in 4.69%, hypertension in 12.5%, and coronary artery disease in 3.13% of the included cases. Patients received antihypertensive drugs: angiotensin-converting enzyme inhibitor or angiotensin receptor antagonist (1.56%) and calcium channel blockers (9.38%). Baseline clinical characteristics of all participants are presented in Table 1.

### 3.2. Echocardiography

Echocardiographic parameters are shown in Table 2. There was no statistically significant difference (*P* > 0.05) between baseline and each follow-up point during chemotherapy.

### 3.3. Electrocardiography

The incidence of abnormal ECG increased from 43.75% at baseline to 65.63% at the end of the treatment (Table 3). This was mainly due to a higher proportion of patients with fQRS after chemotherapy (26.56% to 53.13%, *P* < 0.01).

After three cycles of chemotherapy, heart rate (HR) (76.66 ± 11.99 to 81.23 ± 13.28 bpm, *P*=0.037), QRS dispersion (QTd) (21.25 ± 10.95 to 27.50 ± 13.50 ms, *P* < 0.01), SV1 (1.18 ± 0.41 to 1.42 ± 0.49 ms, *P* < 0.01), and Sokolow–Lyon index (SLI) (1.92 ± 0.59 to 2.26 ± 0.69 mV, *P* < 0.01) increased significantly. At the end of the treatment, HR (76.66 ± 11.99 to 82.14 ± 12.74 bpm, *P*=0.013), P-wave dispersion (Pd) (20.38 ± 9.76 to 16.81 ± 9.41 ms *P*=0.029), corrected QT interval (QTc) (411.38 ± 26.83 to 421.69 ± 21.30 ms, *P*=0.032), T-peak to T-end (TpTe) (73.63 ± 14.20 to 80.13 ± 14.37 ms, *P*=0.024), RR (0.80 ± 0.12 to 0.75 ± 0.11 s, *P*=0.011), SV1 (1.18 ± 0.41 to 1.49 ± 0.48 mV, *P* < 0.01), RV5 (0.74 ± 0.33 to 0.88 ± 0.40 mV, *P*=0.037), SLI (1.92 ± 0.59 to 2.37 ± 0.65 mV, *P* < 0.01), and iCEB (4.29 ± 0.59 to 4.03 ± 0.53, *P*=0.011) deteriorated markedly (all *P* < 0.05) (Table 4).

### 3.4. Receiver Operating Characteristic (ROC) Analysis

**Figure 1:**
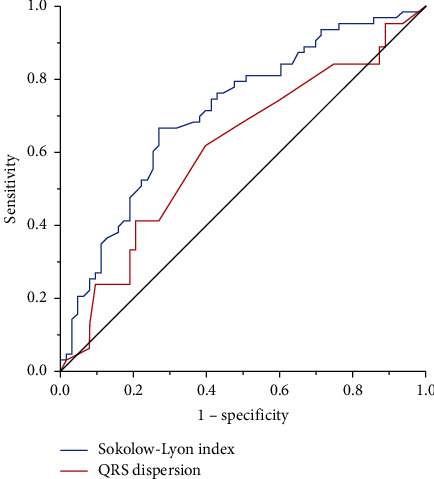
ROC curve for two electrocardiographic parameters to discriminate between pre- and posttherapy.

Table 5 shows the ROC curves generated using two ECG parameters to discriminate between before and after chemotherapy. Compared with the QTd, SLI had a greater area under the ROC curve and a cutoff value with 2.12 had a sensitivity of 67.2% and specificity of 71.9% for differentiating patients after chemotherapy from baselines. For QTd, the area under the ROC curve was 0.61 and a cutoff value with 0.55 had a sensitivity of 60.9% and specificity of 60.9% for differentiating patients after chemotherapy from baselines (Figure 1).

## 4. Discussion

Cardiotoxicity following chemotherapy in patients with breast cancer is a potentially life-threatening complication. Cardiac function can be assessed with echocardiography and cardiac biomarkers. However, there are few studies on electrocardiographic characteristics following chemotherapy in patients with cancer, especially breast cancer. To the best of our knowledge, no study has assessed electrocardiographic parameters immediately after completion of chemotherapy infusion.

Our study used ECGs to evaluate the cardiac electrophysiological changes in patients with breast cancer who received chemotherapy. The main findings of this study are as follows: (1) the incidence of abnormal ECG increased from 43.8% at baseline to 65.6% during follow-up, and this was mainly due to a higher proportion of patients with fQRS; (2) HR, Pd, QTc, TpTe, RR, SV1, RV5, SLI, and iCEB deteriorated markedly along with chemotherapy; and (3) QTd and SLI had high sensitivity and specificity in differentiating patients after therapy from baselines. These findings indicate the development of both depolarization and repolarization abnormalities following chemotherapy.

fQRS is a surrogate marker of myocardial conduction delay or heterogeneity with a prevalence ranging from 1% to 30% of the general population [13–15]. 26.6% (67/252) of breast cancer patients had fQRS after anthracycline-based chemotherapy [16]. At 1-year follow-up, 19 of 52 (37.4%) breast cancer patients receiving locoregional radiotherapy had developed fQRS on ECG [17]. Moreover, the prevalence of fQRS significantly increased in large B-cell lymphoma patients treated with anthracycline-based chemotherapy (15.8% to 28.9%, *P*=0.041) [18]. We found fQRS in 53.1% of breast cancer patients after chemotherapy. In patients with coronary artery disease, fQRS has been shown to be associated with all-cause mortality and cardiac events [19]. Myocardial fibrosis may disrupt QRS morphology and lead to fragmentation of QRS on 12-lead ECG. Chemotherapeutic agents can trigger apoptosis or cause necrotic myocyte death. fQRS occurs when ventricular depolarization (VD) becomes abnormal and has been identified as an ECG biomarker of myocardial fibrosis and can be used to predict adverse cardiovascular events [12, 20].

In the ECG, ventricular repolarization (VR) is represented by QTc intervals, and QTc prolongations relate to a higher risk of ventricular arrhythmias in different conditions. As a risk factor for torsades de pointes (TdP) and sudden cardiac death, QTc prolongation is a toxicity of significant concern [21, 22]. Puppe et al. found a significant increase in QTc intervals after breast cancer treatment with 4 cycles of EC-Doc regimen (epirubicin, cyclophosphamide, and docetaxel) [23]. Several investigations already demonstrated significant QTc prolongation induced by anticancer therapies, especially in anthracycline regimens [24–26]. Similar to our study, in patients with breast neoplasms undergoing chemotherapy regimen with anthracycline (A; doxorubicin), cyclophosphamide (C), and taxane (T; paclitaxel), Veronese et al. also observed prolongation of the QTc interval [27]. In addition, in our study, 25.0% and 31.3% of breast patients were treated with aromatase inhibitor (AI) and tamoxifen (TAM), respectively. Several studies reveal the potential for endocrine therapy to induce ventricular arrhythmias, particularly TdP [28–30].

In this study, our results revealed a significantly increased QTd after chemotherapy. The QTd is defined as the difference between maximal and minimal QT intervals on a 12-lead surface ECG and reflects the regional heterogeneity of VR. Prolonged QT dispersion was even shown to predict acute heart failure in patients after high-dose cyclophosphamide therapy [31]. Further, it has been regarded as an index of ventricular arrhythmia, which may lead to sudden cardiac death [32]. Patients with breast cancer treated with trastuzumab after an anthracycline-based regimen exhibited a significantly higher QTd than nontreated patients (0.064 ± 0.023s vs. 0.051 ± 0.016 s, respectively, *P*=0.034) [33].

SLI is recommended as diagnostic screening method for left ventricular hypertrophy. In this study, we found SLI increased through chemotherapy and thus appears to represent a transitional state from a normal healthy heart to heart failure with preserved ejection fraction. iCEB can provide information about both the depolarization and repolarization phases of the cardiac action potential and is a surrogate marker of excitation wavelength. In experimental studies, a 10% variation (either increase or decrease) of iCEB values from baseline showed to be a promising marker for drug-induced arrhythmic risk [32, 34]. However, data from clinical trials are scarce. To date, there is no comprehensive, easy to measure, and widely available risk marker available. High iCEB values are associated with TdP and low values with non-TdP-mediated VT/VF [10]. In this study, our results revealed a decreased iCEB after chemotherapy in breast cancer. In this study, chemotherapy did not induce a significant change in LVEF. Importantly, LVEF measurement shows a low sensitivity for the early detection of subclinical cardiotoxicity [35]. This might explain why in our observation period we could not detect any decrease in LVEF despite significant electrocardiographic abnormities. Therefore, echocardiography might be suboptimal for detecting acute cardiac complications. These data supported the idea that ECG could identify mild cardiotoxicity in an earlier stage than echocardiography Whilst strain imaging can also be used for early detection of myocardial damage, the advantage of electrocardiography is their rapid and wide availability for routine clinical use. Regular ECG monitoring after initiation of chemotherapy hence is great of importance and may help cardiologists and oncologists tailor treatments during clinical works.

Several limitations of the present study should be noted. Firstly, our study investigated a small number and short follow-up of patients in a single center. Further studies are needed to verify our findings. Secondly, baseline thyroid function and history of heart failure were not collected, which may influence the ECG changes of the patients. Thirdly, the cardiac biomarkers, such as brain natriuretic peptide and troponin, were not tested in most of the included patients because it is limited by medical insurance. Finally, patients in this study are treated with multiple chemotherapeutics such as anthracyclines and cyclophosphamide, which may cause cardiotoxicity that is indistinguishable. Larger prospective studies examining the roles of ECG parameters for risk stratification purposes are needed in the future.

## 5. Conclusions

In this prospective study of patients with breast cancer who underwent chemotherapy, cardiotoxicity can also manifest as the emergence of ECG abnormalities, specifically abnormal ventricular repolarization. With further study, SLI and QTd ratio could potentially be used for differentiating patients after therapy from baselines. The data from this study demonstrated that ECG can be conducted to evaluate the subclinical cardiac damage for breast cancer patients after chemotherapy. ECG could help to detect subclinical cardiac dysfunction earlier than echocardiography. Regular ECG monitoring may help to detect early cardiotoxicity during follow-up following chemotherapy.

## Figures and Tables

**Table 1:** Baseline clinical characteristics and cardiovascular risk factors in breast cancer patients.

Characteristics	
Age, years, mean ± SD	49.09 ± 9.61
Females, *n* (%)	64 (100)
BMI, kg/m^2^	24.02 ± 3.18
Systolic pressure, mmHg	124.91 ± 14.86
Diastolic pressure, mmHg	81.63 ± 11.01

Medical history, *n* (%)	
Hypertension	8 (12.50)
Diabetes mellitus	3 (4.69)
Dyslipidemia	0 (0)
Coronary heart disease	2 (3.13)

Smoking, *n* (%)	
Current smoker	0 (0)
Former smoker	0 (0)
Nonsmoker	63 (100)
HR status, *n* (%)	
ER− and PR−	27 (42.19)
ER+ and/or PR+	36 (56.25)
HER-2+	24 (37.50)
Histology type, *n* (%)	
Ductal carcinoma	3 (4.69)
Lobular carcinoma	59 (92.19)
DCIS	2 (3.13)

Cancer stage, *n* (%)	
I	23 (35.94)
II	37 (57.81)
III	4 (6.25)
IV	0 (0)

Surgery, *n* (%)	
Lumpectomy	41 (64.06)
Mastectomy	23 (35.94)
Cardiovascular medications, *n* (%)	
Beta-blockers	0 (0)
Calcium channel antagonist	6 (9.38)
Antiplatelet medicines	1 (1.56)
ACEI/ARBs	1 (1.56)
Statins	0 (0)

Cancer therapy, *n* (%)	
Anthracycline	49 (76.56)
Taxane	60 (93.75)
Anti-HER2	21 (32.81)
Anthracycline and anti-HER2	15 (23.44)

Cumulative dose of anthracycline, mg	
Median (range)	354.29 ± 149.22
<430	37 (57.81)
≥430	12 (18.75)

Cumulative dose of taxane, mg	
Median (range)	771.00 ± 345.93
<760	40 (62.50)
≥760	20 (31.25)

Endocrine therapy, *n* (%)	
AI	16 (25.00)
TAM	20 (31.25)
None	28 (43.75)

BMI, body mass index; ACEIs, angiotensin-converting enzyme inhibitors; ARBs, angiotensin receptor blockers; HER2, human epidermal growth factor receptor 2; DCIS, ductal carcinoma in situ; AI, aromatase inhibitor; BMI, body mass index; DCIS, ductal carcinoma in situ; ER, estrogen receptor; HR, hormone receptor; PR, progesterone receptor; TAM, tamoxifen.

**Table 2:** Echocardiographic parameters before and at each follow-up point during chemotherapy in breast cancer patients.

Variable	T0	T1	*P* value	T2	*P* value	T3	*P* value
LVIDd (cm)	4.60 ± 0.29	4.64 ± 0.29	0.422	4.67 ± 0.30	0.184	4.58 ± 0.27	0.711
LA (cm)	3.00 ± 0.31	3.05 ± 0.39	0.458	3.06 ± 0.27	0.425	3.01 ± 0.32	0.919
LVEF (%)	67.00 ± 4.07	66.09 ± 3.94	0.201	65.95 ± 3.96	0.139	65.34 ± 4.00	0.052
E/A ratio	1.10 ± 0.35	1.16 ± 0.48	0.389	1.11 ± 0.41	0.873	1.03 ± 0.38	0.334
E/E′ ratio	7.64 ± 1.74	7.99 ± 1.99	0.298	7.86 ± 1.93	0.519	7.28 ± 1.96	0.310

Values are mean ± SD. ^*∗*^Compared with T0 *p* < 0.05. T0, baseline before chemotherapy; T1, after 1 cycle of chemotherapy; T2, after 3 cycles of chemotherapy; T3, end of chemotherapy; LVIDd, left ventricular internal dimension diastole; LA, left atrial diameter; LVEF, left ventricle ejection fraction.

**Table 3:** ECG changes before and at each follow-up point during chemotherapy in BC patients.

ECG changes	T0	T1	*P* value	T2	*P* value	T3	*P* value
Abnormal ECG, *n* (%)	28 (43.75)	36 (56.25)	0.157	41 (64.06)	0.021^*∗*^	42 (65.63)	0.013^*∗*^
ST-T changes, *n* (%)	7 (10.94)	10 (15.63)	0.435	10 (15.62)	0.435	10 (15.62)	0.435
ST changes	6 (9.38)	7 (10.94)	0.770	7 (10.94)	0.770	7 (10.94)	0.770
T-wave changes	1 (1.56)	6 (9.38)	0.052	8 (12.50)	0.016^*∗*^	6 (9.38)	0.052
Arrhythmias, *n* (%)	13 (20.31)	11 (17.19)	0.651	13 (20.31)	1.000	14 (21.88)	0.828
Sinus tachyarrhythmia	1 (1.56)	3 (4.69)	0.310	5 (7.81)	0.094	6 (9.38)	0.052
Ventricular premature beats	2 (3.13)	2 (3.13)	1.000	2 (3.13)	1.000	3 (4.69)	0.310
First-degree AVB	3 (4.69)	2 (3.13)	0.648	1 (1.56)	0.310	1 (1.56)	0.310
Intraventricular block	1 (1.56)	1 (1.56)	1.000	1 (1.56)	1.000	1 (1.56)	1.000
QTc prolongation, *n* (%)	3 (4.69)	4 (6.25)	0.697	1 (1.56)	0.310	2 (3.13)	0.648
fQRS, *n* (%)	17 (26.56)	27 (42.19)	0.063	30 (46.88)	0.017^*∗*^	34 (53.13)	<0.01^*∗*^

Values are mean ± SD. ^*∗*^Compared with T0, *p* < 0.05. T0, baseline before chemotherapy; T1, after 1 cycle of chemotherapy; T2, after 3 cycles of chemotherapy; T3, end of chemotherapy; AVB, atrioventricular block; QTc, corrected QT interval; fQRS, fragmented QRS.

**Table 4:** Electrocardiographic parameters before and at each follow-up point during chemotherapy in BC patients.

Variables	T0	T1	*P* value	T2	*P* value	T3	*P* value
HR (bpm)	76.66 ± 11.99	78.83 ± 11.30	0.321	81.23 ± 13.28	0.037^*∗*^	82.14 ± 12.74	0.013^*∗*^
PWA (mV)	0.11 ± 0.03	0.12 ± 0.03	0.454	0.12 ± 0.04	0.623	0.12 ± 0.04	0.438
PWD (ms)	95.53 ± 12.05	95.61 ± 11.60	0.973	94.06 ± 11.87	0.529	96.19 ± 16.58	0.779
PRI (ms)	148.97 ± 20.37	148.05 ± 21.29	0.835	147.78 ± 21.54	0.788	149.58 ± 34.16	0.890
Pd (ms)	20.38 ± 9.76	18.38 ± 9.66	0.218	18.31 ± 8.75	0.204	16.81 ± 9.41	0.029^*∗*^
QRS axis (°)	39.33 ± 30.13	37.83 ± 30.28	0.769	40.29 ± 24.74	0.853	37.49 ± 29.51	0.720
QRSD (ms)	87.05 ± 13.88	87.02 ± 12.88	0.989	89.14 ± 12.66	0.371	91.64 ± 13.43	0.050
QTc (ms)	411.38 ± 26.83	415.61 ± 26.67	0.378	414.94 ± 32.45	0.458	421.69 ± 21.30	0.032^*∗*^
QTd (ms)	21.25 ± 10.95	24.75 ± 11.92	0.102	27.50 ± 13.50	<0.01^*∗*^	24.94 ± 11.71	0.085
TpTe (ms)	73.63 ± 14.20	76.31 ± 18.64	0.386	76.69 ± 21.64	0.323	80.13 ± 14.37	0.037^*∗*^
RR (s)	0.80 ± 0.12	0.78 ± 0.11	0.252	0.76 ± 0.12	0.042	0.75 ± 0.11	0.011^*∗*^
SV1 (mV)	1.18 ± 0.41	1.30 ± 0.41	0.144	1.42 ± 0.49	<0.01^*∗*^	1.49 ± 0.48	<0.01^*∗*^
RV5 (mV)	0.74 ± 0.33	0.79 ± 0.38	0.512	0.84 ± 0.42	0.158	0.88 ± 0.40	0.037^*∗*^
SLI (mV)	1.92 ± 0.59	2.08 ± 0.61	0.151	2.26 ± 0.69	<0.01^*∗*^	2.37 ± 0.65	<0.01^*∗*^
iCEB	4.29 ± 0.59	4.26 ± 0.54	0.781	4.10 ± 0.62	0.069	4.03 ± 0.53	0.011^*∗*^

Values are mean ± SD. ^*∗*^Compared with T0, *p* < 0.05. T0, baseline before chemotherapy; T1, after 1 cycle of chemotherapy; T2, after 3 cycles of chemotherapy; T3, end of chemotherapy; HR, heart rate; PWA, P-wave amplitude; PWD, P-wave duration; PRI, PR interval; Pd, P-wave dispersion; QRSD, QRS duration; QTc, corrected QT interval; QTd, QRS dispersion; TpTe, T-peak to T-end; SLI, Sokolow–Lyon index; iCEB: index of cardioelectrophysiological balance.

**Table 5:** ROC curve analyses of electrocardiographic parameters.

Variable	AUC	95% CI	Cutoff value	Sensitivity	Specificity
SLI	0.710	0.620–0.799	2.12	0.672	0.719
QTd	0.606	0.507–0.704	0.55	0.609	0.609

ROC, receiver operating characteristic; AUC, area under the curve; CI, confidence interval; SLI, Sokolow–Lyon index; QTd, QRS dispersion.

## Data Availability

The data used to support the findings of this study are available from the corresponding author upon request.
